# Analysis of brief language tests in the detection of cognitive decline and dementia

**DOI:** 10.1590/S1980-57642008DN10100007

**Published:** 2007

**Authors:** Marcia Radanovic, Maria Teresa Carthery-Goulart, Helenice Charchat-Fichman, Emílio Herrera Jr., Edson Erasmo Pereira Lima, Jerusa Smid, Cláudia Sellitto Porto, Ricardo Nitrini

**Affiliations:** 1 MD, MSc, PhD in Neurology, Behavioral and Cognitive Neurology Unit, Department of Neurology, University of São Paulo School of Medicine, São Paulo, Brazil.; 2 MSc in Neuroscience, PhD in Neurology, Behavioral and Cognitive Neurology Unit, Department of Neurology, University of São Paulo School of Medicine, São Paulo, Brazil.; 3 MSc, PhD in Neuroscience, Behavioral and Cognitive Neurology Unit, Department of Neurology, University of São Paulo School of Medicine, São Paulo, Brazil.; 4 MD, Department of Internal Medicine, Catanduva School of Medicine, Catanduva, Brazil.; 5 Post Graduate Student, Behavioral and Cognitive Neurology Unit, Department of Neurology, University of São Paulo School of Medicine, São Paulo, Brazil.; 6 MSc, PhD in Neurology, Behavioral and Cognitive Neurology Unit, Department of Neurology, University of São Paulo School of Medicine, São Paulo, Brazil.; 7 MD, PhD, Behavioral and Cognitive Neurology Unit, Department of Neurology, and Cognitive Disorders Reference Center (CEREDIC). Hospital das Clínicas of the University of São Paulo School of Medicine, São Paulo, Brazil.

**Keywords:** language tests, cognitive disorders, dementia, Alzheimer disease, education, testes de linguagem, transtorno cognitivo, demência, doença de Alzheimer, escolaridade

## Abstract

Lexical access difficulties are frequent in normal aging and initial stages of dementia.Verbal fluency tests are valuable to detect cognitive decline, evidencing lexico-semantic and executive dysfunction.

**Objectives:**

To establish which language tests can contribute in detecting dementia and to verify schooling influence on subject performance.

**Method:**

74 subjects: 33 controls, 17 Clinical Dementia Rating (CDR) 0.5 and 24 (Brief Cognitive Battery - BCB e Boston Naming Test - BNT) 1 were compared in tests of semantic verbal fluency (animal and fruit), picture naming (BCB and BNT) and the language items of Mini Mental State Examination (MMSE).

**Results:**

There were significant differences between the control group and both CDR 0.5 and CDR 1 in all tests. Cut-off scores were: 11 and 10 for animal fluency, 8 for fruit fluency (in both), 8 and 9 for BCB naming. The CDR 0.5 group performed better than the CDR 1 group only in animal fluency. Stepwise multiple regression revealed fruit fluency, animal fluency and BCB naming as the best discriminators between patients and controls (specificity: 93.8%; sensitivity: 91.3%). In controls, comparison between illiterates and literates evidenced schooling influence in all tests, except for fruit fluency and BCB naming. In patients with dementia, only fruit fluency was uninfluenced by schooling.

**Conclusion:**

The combination of verbal fluency tests in two semantic categories along with a simple picture naming test is highly sensitive in detecting cognitive decline. Comparison between literate and illiterate subjects shows a lesser degree of influence of schooling on the selected tests, thus improving discrimination between low performance and incipient cognitive decline.

## Abstract (pt)

Dificuldades de acesso lexical são freqüentes no envelhecimento normal e fases iniciais de demência.

Testes de fluência verbal são úteis para detectar declínio cognitivo, evidenciando disfunções léxicosemânticas e executivas.

**Objetivos:**

Estabelecer quais testes de linguagem podem contribuir para detectar demência e verificar a influência da escolaridade no desempenho dos sujeitos.

**Métodos:**

74 indivíduos: 33 controles, 17 CDR 0,5 e 24 CDR 1, foram comparados quanto a fluência verbal semântica (animais e frutas), nomeação de figuras (BCB e TNB) e provas de linguagem do MEEM.

**Resultados:**

Houve diferenças significantes em todas as provas entre controles e os grupos CDR 0,5 e CDR 1. As notas de corte foram: 11 e 10 para animais, 8 (para ambos) para frutas, 8 e 9 para nomeação da BCB. O grupo CDR 0,5 teve desempenho superior ao CDR 1 apenas na fluência de animais. Fluência de frutas, de animais e nomeação da BCB foram as variáveis que melhor discriminaram pacientes e controles (especificidade: 93,8%; sensibilidade: 91,3%). Em controles, a comparação entre analfabetos e alfabetizados evidenciou influência da escolaridade em todas as provas, com exceção da fluência de frutas e nomeação da BCB. Em pacientes com demência só a fluência de frutas não sofreu influência da escolaridade.

**Conclusão:**

A combinação de testes de fluência verbal em duas categorias semânticas e nomeação de figuras simples é altamente sensível para detectar declínio cognitivo. A comparação entre alfabetizados e analfabetos mostrou menor influência da escolaridade nos testes selecionados, facilitando a discriminação entre baixo desempenho e declínio cognitivo incipiente.

The frequency of language disturbances in Alzheimer's disease (AD) depends on the disease severity, ranging from 36% to 100% in mild to severe cases, respectively^1^. The classic descriptions of language disturbances in AD include marked alterations in lexico-semantic aspects with relative preservation of the phonologicalsyntactic aspects, the latter remaining rare until the most advanced stages of the disease^2-4^.

Worsening of language disturbances can be correlated to cognitive deterioration^5^ as determined by the Global Deterioration Scale (GDS)^6^. However, until advanced stages, the individual can maintain communicative abilities such as simple conversations, in spite of reduced initiative for communication and spontaneous speech, vocabulary limitation, or a reduced capacity to link ideas and convey precise information. A recent study from Bayles et al.^7^ highlights the permanence of linguistic abilities related to the implicit system, such as repetition, recognition of one's own name and social abilities even in the more severe stages of the disease. Lexical and semantic abilities in AD are studied mostly through visual confrontation naming and semantic verbal fluency tests. Although most researchers agree that AD patients have difficulties in these tasks in the initial stages of disease^3^, there is no consensus on the nature of these deficits.

Some authors hypothesize a deterioration in the “semantic store”^8-10^ while others interpret the difficulties as being attributable to an impaired access to this store^11^. After a review of the literature on the subject, Kempler^3^ suggests that the naming difficulties cannot be explained by a single factor alone. Visuospatial deficits, as well as deficits in attention, lexical access and semantic representations can all account for these difficulties. The assumption that semantic deterioration initiates with loss of knowledge of specific attributes of concepts, progressively affecting the recognition of their semantic categories has not yet been confirmed. Martin and Fedio^9^ agree with this hypothesis but Nebes and Brady^11^ propose that, in tasks with lower demands on memory and attention (for example, when patients are asked whether a given attribute is related to a concept, instead of having to decide among several attributes related to the same target), AD patients show preservation of the semantic knowledge related to object categories and attributes.

Verbal fluency tasks are sensitive in detecting ageassociated changes, because they involve lexical access, speed of information processing and working memory^1,8,12-16^. In verbal fluency tasks, AD patients in initial stages show better performance when asked to generate items according to formal criteria (words that begin with the letter "F", for example) than when they have to generate items according to semantic categories (animal or fruits, for example)^17^. Variables such as education can determine lower cut-off scores and differences in searching strategies^16,18^. Faber-Langendoen et al.^1^ reports that verbal fluency tasks evaluate not only language but also other cognitive processes, such as executive functions. From this perspective, Chertkow and Bub^8^ interpret the difficulties in AD patients as a result of not only semantic deterioration, but also impairment in search strategies. Della Sala et al.^15^ agree with this hypothesis and highlight the executive component of this task, making the task more sensitive for AD diagnosis.

From the neuropsychological perspective, some of the other forms of dementia can be considered as a group, due to similarities in their profiles of cognitive deterioration. This holds true for the group of “subcortical dementia”, which include, among other etiologies, Parkinson’s disease (PD) with dementia, Huntington’s disease (HD) and some clinical forms of vascular dementia (VD). Although language abilities are relatively well preserved in subcortical dementias (when compared to cortical dementias), verbal fluency is impaired, both in semantic and formal categories. (Formal fluency is a task requiring the production of words beginning with a given letter). A relatively poor performance in the formal rather than semantic category is considered a typical finding in subcortical dementia. Executive dysfunction, one of the hallmarks of subcortical dementia, is thought to play a major role in this impairment considered less attributable to semantic deterioration, at least in the early-to-moderate stages of this type of dementia^19,20^. Visual confrontation naming is also relatively preserved in subcortical dementias when compared to AD, and impairment in naming can be due to several causes, including visuospatial deficits (especially perceptual), difficulties in lexical access, or impairment in the phonological / motor output^21^.

There is a substantial group of patients that pose a challenge to most clinicians: those complaining of “forgetfulness”, “difficulties in finding the appropriate words” and exhibiting mild cognitive deficits when tested, yet do not fulfill the criteria for a diagnosis of dementia. They may remain in this condition for a variable period of time, and finally develop a full clinical picture of dementia. This condition, described as “mild cognitive impairment” (MCI)^22^ can sometimes be very difficult to differentiate from age-related cognitive decline or even from normal subjects^23^, especially when the patients have low educational level.

Educational level poses one of the greatest obstacles in using language tests to diagnose dementia and MCI. Illiteracy compromises all tasks that require reading, writing and spelling, and reduces the possibility of assessing actual cognitive decline. Reading habits influence the acquisition of knowledge and this also has an impact on the subject’s performance in comprehension tasks (even in the oral modality). In addition, most language tests used in Brazil were developed in English-speaking countries, and had to be translated and adapted to Portuguese.

The objectives of our study were:

a) to establish which combination of selected language tests can contribute to detecting dementia;
b) to verify the influence of schooling on the performance of different subgroups (controls, MCI and mild dementia) on these language tests.

## Methods

We studied 74 subjects: 33 controls, 17 with mild cognitive impairment (MCI – CDR 0.5) and 24 patients with mild dementia (CDR 1). The diagnosis of dementia was established according to the DSM-IV criteria^24^, based on clinical and neuropsychological evaluation that included physical and neurological examination, the Pfeffer Functional Activities Questionnaire (PFAQ)^25^ score >5 and Mini Mental State Examination (MMSE)^26^ below specific education-adjusted scores (27 for subjects with educational level >7 years, 24 for those with 1 to 7 years of schooling, and 19 for the illiterate)^27^. The evaluation also contained the Brazilian version of the CERAD battery^28^, and the Brief Cognitive Battery (BCB)^29^ including immediate and delayed recall of 10 simple objects presented as line drawings. Participants were considered illiterates when fulfilling all of the following three conditions: they had never attended school or had attended for less than 1 year, they considered themselves unable to read, and they were unable to read the phrase ‘‘close your eyes’’ from the MMSE.

The language examination included verbal fluency (animals and fruits), naming of 10 pictures from the BCB, naming of 15 pictures from the Boston Naming Test (BNT)^30^ (which are inserted in the CERAD battery) and the language tasks from the MMSE. The full description of the language tests is shown in the Appendix.

Dementia severity was rated using the Clinical De-mentia Rating Scale (CDR)^31,32^. The clinical type of dementia was established according to the National Institute of Neurological and Communicative Disorders and Stroke-Alzheimer’s Disease and Related Disorders Association (NINCDS-ADRDA)^33^ criteria for AD; National Institute of Neurologic Disorders and Stroke- Association Internationale pour la Recherche et L’Enseigment en Neurosciences (NINDS-AIREN) criteria for vascular dementia and for AD with cerebrovascular disease (AD+CVD)^34^, and the United Kingdom Parkinson’s Disease Society Brain Bank criteria for Parkinson’s disease^35^. The etiology of the dementia in the patient group was as follow: 16 subjects had probable AD, four had probable vascular dementia, two had Parkinson’s disease with dementia, one had Huntington’s disease, and one patient an underlying condition which could not be determined.

Subjects having difficulty performing the cognitive tests due to auditory, visual, or other physical problems which could have affected their performance were excluded from the study. Subjects with PFAQ score <6 and MMSE above the specific education-adjusted scores were selected as a control group. These subjects were evaluated using the same protocol. All groups were matched for age, schooling and gender.

This study was approved by the Ethics Committee of the Hospital das Clínicas,University of São Paulo School of Medicine, Brazil. Informed consent was obtained from all participants or from a family member, when appropriate.

### Statistical analysis

The dementia and control groups were compared using non-parametric Analysis of Variance (Kruskal- Wallis with Dunn’s post-test) in the following tests: verbal fluency (animal and fruits), naming of 10 pictures from the BCB, naming of 15 pictures from the BNT and the language tasks from the MMSE.

The cut-off scores which best discriminated the groups were determined through receiver operating characteristic (ROC) analysis for each variable, establishing which of these variables could most accurately detect MCI and dementia. Stepwise multiple regression was performed to verify which combination of simple language tasks could most significantly contribute toward building a predictive model for the diagnosis of dementia. All analyses were performed using the statistical software SPSS® version 10.0.

## Results

The demographic variables of the sample are displayed in Table 1. The gender distribution was CDR 0: 14 F/19 M; CDR 0.5: 10 F/7 M; CDR 1: 15 M/9 F (p= 0.279). There were significant differences between controls and the other groups (CDR 0.5 and 1) in all language tasks (p<0.01). The CDR 0.5 group had significantly superior performance compared to that observed in the dementia group on the fluency tasks (p<0.01) (Table 1).

**Table 1:** Demographic variables and performance of subjects in the language tests according to CDR classification.

Variable	CDR 0		CDR 0.5		CDR 1		p	Intergroup difference
	M (SD)	Range	M (SD)	Range	M (SD)	Range		
Age	76.7 (5)	71-91	76.9 (6.6)	65-88	79.3 (5.4)	71-92	0.177	-
Schooling	1.8 (2.7)	0-10	0.4 (0.8)	0-3	1.7 (3.3)	0-11	0.169	-
MMSE total	23 (3.4)	19-30	17.6 (2)	15-20	16 (2.9)	11-22	<0.0001	All groups differ
MMSE language items	6.6 (1.1)	5-8	5.5 (0.9)	3-7	5.7 (1.3)	2-8	0.003	0 & 0.5; 0 & 1
BNT	10.3 (2.5)	5-15	8.3 (2.1)	4-12	7.8 (2.5)	3-13	0.0009	0 & 0.5; 0 & 1
BCB naming	9.6 (0.7)	7-10	8.4 (1.8)	3-10	7.8 (1.4)	5-10	<0.0001	0 & 0.5; 0 & 1
Animal fluency	12.9 (3.5)	6-20	10.2 (3)	7-17	7 (2.6)	2-12	<0.0001	All groups differ
Fruit fluency	10.3 (2.1)	6-14	7.6 (1.8)	3-10	5.8 (2.5)	0-10	<0.0001	All groups differ

M, mean; SD, standard-deviation; BCB, brief cognitive battery; BNT, Boston naming test; CDR, clinical dementia rating; MMSE, mini mental state examination.

In the control group, there were significant differences between illiterates and subjects with one or more years of education in the animal fluency, BNT and language tasks of MMSE. Among the patients with dementia, there were significant differences between illiterates and subjects with one or more years of education in all tasks, except fruit fluency (Table 2).

**Table 2:** Performance of subjects in the language tests according to literacy.

	Literates	Illiterates	
Language test	M (SD)	M (SD)	p
***CDR 0***			
MMSE language items	7.64 (0.6)	5.62 (0.5)	<0.0001
BNT	11.23 (2.56)	9.43 (2.15)	0.03
BCB naming	9.75 (0.57)	9.43 (0.89)	0.24
Animal fluency	14.23 (3.38)	11.5 (3.14)	0.013
Fruit fluency	10.52 (1.94)	10 (2.39)	0.52
***CDR 1***			
MMSE language items	6.5 (1)	5.21 (1.18)	0.015
BNT	9.8 (1.98)	6.35 (1.86)	0.001
BCB naming	8.6 (0.96)	7.21 (1.42)	0.015
Animal fluency	8.61 (1.95)	5.85 (2.4)	0.01
Fruit fluency	6.3 (2.4)	5.57 (2.65)	0.65

M, mean; SD, standard-deviation; BCB, brief cognitive battery; BNT, Boston naming test; CDR, clinical dementia rating; MMSE, mini mental state examination.

The tasks that best discriminated between CDR 0 and CDR 1 groups were animal fluency, fruit fluency and naming of BCB pictures. The best discrimination between CDR 0 and CDR 0.5 groups was achieved through the fruit fluency and naming of BCB pictures tasks. Discrimination between CDR 0.5 and CDR 1 groups was poor, and the animal fluency task provided the best sensitivity and specificity (Table 3). The language tasks of the MMSE were not useful in discriminating between any combination of groups.

**Table 3:** Cut off scores, sensitivity and specificity for each variable in the discrimination of CDR subgroups.

Variable	Cut-off score	Sensitivity (%)	Specificity (%)	AUC	95% CI AUC
***CDR 0 X CDR 1***					
BNT	9	79.2	70.6	0.76	0.63 to 0.86
BCB naming	9	95.8	71.9	0.88	0.77 to 0.95
Animal fluency	10	95.8	72.7	0.91	0.8 to 0.97
Fruit fluency	8	87.5	81.8	0.91	0.8 to 0.97
***CDR 0 X CDR 0.5***					
BNT	9	70.6	60.6	0.73	0.59 to 0.84
BCB naming	9	68.7	71.9	0.74	0.6 to 0.85
Animal fluency	11	76.5	72.7	0.72	0.58 to 0.84
Fruit fluency	8	68.7	81.8	0.82	0.68 to 0.91
***CDR 0.5 X CDR 1***					
BNT	7	54.2	64.7	0.57	0.40 to 0.72
BCB naming	9	95.8	31.2	0.63	0.47 to 0.68
Animal fluency	6	45.8	100	0.78	0.63 to 0.89
Fruit fluency	5	37.5	93.7	0.71	0.55 to 0.84

AUC, area under the curve; CI, confidence interval; BCB, brief cognitive battery; BNT, Boston naming test; CDR, clinical dementia rating.

In the stepwise multiple regression analysis, the variable fruit fluency, animal fluency and BCB naming were the tasks which best contributed toward the generation of a model that discriminated patients and controls with 93.8% of specificity and 91.3% of sensitivity (Table 4).

**Table 4:** Stepwise multiple regression analysis results.

Selected variables	Actual group CDR		Predicted CDR	% of correct classification
		0 1		
Fruit	0	26 6		81.3
	1	616		72.7
	accuracy			72.7
Fruit + animal	0	29 3		90.6
	1	319		86.4
	accuracy			88.9
Fruit + animal + BCB	0	30 2		93.8
	1	220		90.9
	accuracy			92.6

BCB, brief cognitive battery; CDR, clinical dementia rating.

## Discussion

The clinical diagnosis of dementia still poses challenges, the most notable of which include: how to obtain an accurate diagnosis without submiting the patient to a tiring extensive neuropsychological battery, often inaccessible to a large proportion of the population (in primary care assistance and non-specialized centers, for example)? How to establish this diagnosis in populations that are extremely heterogeneous regarding formal education level and cultural aspects, such as the Brazilian population, taking into account the critical influence that these two factors have on the performance of subjects in neuropsychological tests? How to diagnose dementia in illiterate populations, considering that illiteracy has a large impact on cerebral organization itself^36^?

Thus, health professionals assisting populations at risk of developing dementia have shown increasing interest in obtaining cognitive tests which are easily applied and offer good diagnostic accuracy.Moreover, the development of the concept of MCI poses the additional challenge of trying to identify subjects that already present some degree of cognitive decline yet not fulfilling the clinical criteria for dementia, who might in the future benefit from early treatment (pharmacological treatment, for example) before developing full dementia.

Verbal fluency tasks, both in semantic (like animal) and formal categories (such as letters, FAS) are part of most neuropsychological batteries, due to their reliability as instruments for the mensuration of semantic memory and executive function (developing strategies for information retrieval)^37^. Verbal fluency can be impaired in individuals with MCI, indicating that the degradation of semantic association links is already present at very early stages in individuals at risk of developing AD^38,39^. This finding was replicated in our study. Besides, test-retest situations in this kind of task are also useful to identify patients still in preclinical stages of dementia, because they have a learning behavior profile approaching that encountered in AD patients^40^. AD patients usually present a greater impairment in semantic rather than formal fluency, reflecting the greater damage in medial temporal structures^41,42^.

Comparing individuals with mild dementia according to literacy (literate versus illiterate), we verified that the only task not allowing discrimination between groups is fruit fluency. This seems to indicate that the strong frequency effect of the category is powerful enough to compensate for the executive deficit of these patients, which already exists, as can be verified by low performance in the animal fluency task. Among the variables that are known to influence verbal fluency results are the item frequency and its age of acquisition in the vocabulary^43^. The type of semantic category and its size (number of items available and learned for that category) also influence the performance of patients with AD^44^. Studies in Spanish and English also verified the effectiveness of the use of alternative semantic categories, with the purpose of improving diagnostic reliability^45,46^.

Among the patients that had non-AD dementias, three had some form of subcortical dementia (PD or HD), and four had VD. Considering that about 50% of VD cases present clinically as subcortical dementia and that even in cortical forms of VD the executive dysfunction is highly prominent^47^, we chose to consider these non-AD patients as a single group presenting as subcortical dementia. From this perspective, it is our opinion that a combination of executive (verbal fluency) and semantic (naming) tasks might increase the probability of detection of cognitive impairment when neither the existence nor the etiology of the dementia are *known a priori*, as is frequently the case in primary care assistance.

In the low educated population, we found that the cut-off score (10 animals) that discriminated controls from dementia patients was lower than that usually used in clinical studies in the Brazilian population (12 animals) ^48^. The fact that the fruit category appeared to be less influenced by schooling can be observed by the greater specificity of this task in discriminating between CDR 0 and CDR 1 subjects, when compared to animal fluency (Table 4). Similarly, we found that the naming of pictures of the BCB, when compared to the BNT, allowed better discrimination between CDR 0 and CDR 1 subjects, being more suitable for low educated individuals. Visual confrontation naming tasks are also sensitive to the deterioration of the semantic store system, demonstrating a greater degree of alteration than the phonemic fluency tasks in a meta-analysis of AD patients^49^.

Regarding discrimination between controls and MCI, and between MCI and mild dementia, we observed a decline in the accuracy of diagnoses. The fruit fluency has good specificity for discrimination between CDR 0 and CDR 0.5, with low sensitivity. Visual confrontation naming tests alone are not good at discriminating MCI^50^.

In our study, the discrimination between controls and dementia patients was more accurate than between MCI and dementia patients, as often described in the literature, regardless of the kind of test used. Patients with MCI constitute an extremely heterogeneous population regarding the degree and profile of cognitive loss, making it difficult to separate them from normal individuals by means of short batteries or batteries that emphasize only one cognitive domain. Comparing the normal individuals according to literacy (literate versus illiterate), we verified differences in performance in animal fluency, in the BNT naming and in the language tasks of MMSE. The BNT is an English language test built with stimuli from a culture that is different from ours, and this certainly influenced the results, especially in low educated subjects. The language tasks of the MMSE include reading and writing, which can explain the differences between literate and illiterate. The fruit fluency and BCB naming appear to be less subject to the schooling effect.

The language items of the MMSE were not useful in discriminating the three groups. This may be due to:

a) the low complexity of these tasks: naming of only two items of high frequency (“pencil” and “watch”), reading of a very simple sentence (“Close your eyes”);
b) the fact that some tasks involve abilities that may be preserved until the moderate to severe stages of dementia, such as repetition and writing (the sentence may be more, or less complex depending on the residual capacity of the patient, but still considered correct).

Stepwise multiple regression showed that the combination of two semantic categories associated to a simple task of picture naming can significantly improve discrimination between normal subjects and patients with mild dementia, in low educated individuals. The combination of these two tasks can be effective enough in detecting problems in lexical access and in the generation of search strategies in semantic categories, constituting early markers of cognitive decline. The time taken to administer this combination of tasks does not exceed five minutes and does not require any specific training. The results involving literate and illiterate suggest that fruit fluency and BCB naming might be useful in discriminating between poor performance and incipient cognitive declines in low educated subjects, as these tasks appear to be less influenced by schooling.

In conclusion, the results of this study show that rapid to administrate, straightforward tests can be accurate enough for use in everyday practice. Clinicians often face the difficult task of examining low educated patients and having to decide whether their low performance is attributable to cognitive impairment itself or to a less favorable cultural background, hence the importance of developing new combination of tests as little influenced by schooling as possible. The main limitations of this study are:

a) the small number of subjects in each group;
b) the inclusion of different etiologies of dementia (each type having distinct neuropsychological profiles) in the sample;
c) formal fluency was not assessed.

Future directions for this research include the administration of our brief language battery in a larger population (with the addition of formal fluency assessment), and the comparison of distinct patterns of behavior among the different etiologies of dementia.

## Appendix. Language tests.

- Semantic fluency: the patient is asked to produce a list of all the animals / fruits he / she can remember in one minute. (One minute is given for each category).
- BNT Naming (CERAD version): name the following pictures, presented as line drawings:
  - CAMA (BED)
  - ÁRVORE (TREE)
  - ESCOVA DE DENTES (TOOTHBRUSH)
  - CAMELO (CAMEL)
  - REDE (HAMMOCK)
  - CASA (HOUSE)
  - FLOR (FLOWER)
  - VULCÃO (VOLCANO)
  - CANOA (CANOE)
  - FUNIL (FUNNEL)
  - APITO (WHISTLE)
  - DOMINÓ (DOMINOES)
  - MÁSCARA (MASK)
  - GAITA (HARMONICA)
  - PINÇA (TONGS)
- Language tasks of the MMSE:
  - Name a pencil and a watch shown by the examiner.
  - Repeat “nem aqui, nem ali, nem lá” (equivalent to “no ifs, ands or buts”).
  - Follow a three-stage command: “Pegue este papel com sua mão direita, dobre ao meio e coloque no chão” (“Take a paper in your right hand, fold it in half, and put it on the floor).
  - Read and obey: “Feche os olhos” (“Close your eyes”).
  - Write a sentence.
  - Copy two intersected pentagons
- BCB Naming: name the following 10 pictures, presented as line drawings:
  - SAPATO (SHOE)
  - CASA (HOUSE)
  - PENTE (COMB)
  - CHAVE (KEY)
  - AVIÃO (AIRPLANE)
  - TARTARUGA (TURTLE)
  - BALDE (BUCKET)
  - LIVRO (BOOK)
  - COLHER (SPOON)
  -ÁRVORE (TREE)
